# Post-thaw ATP supplementation enhances cryoprotective effect of iodixanol in rat spermatozoa

**DOI:** 10.1186/s12958-016-0141-5

**Published:** 2016-01-29

**Authors:** Suhee Kim, Sarah Hooper, Cansu Agca, Yuksel Agca

**Affiliations:** Department of Veterinary Pathobiology, College of Veterinary Medicine, University of Missouri-Columbia, 1600 East Rollins Street, Room W191, Columbia, MO 65211 USA

**Keywords:** ATP, Cryopreservation, Rat spermatozoa, Iodixanol

## Abstract

**Background:**

Successful cryopreservation of rat spermatozoa from various strains still remains a challenge. The objective of this study was to determine if combinations of OptiPrep™ (iodixanol) and adenosine 5′-triphosphate (ATP) can improve rat sperm function during the cryopreservation procedure.

**Methods:**

Epididymal rat spermatozoa were frozen under different OptiPrep™ concentrations (0, 1, 2, 3 or 4 %) and were diluted with media supplemented with or without 2 mM ATP after thawing. Post-thaw sperm motility, acrosomal membrane integrity (AMI) and mitochondrial membrane potential (MMP) were then evaluated. In addition, the effect of different OptiPrep™ concentrations on fresh and cooled rat spermatozoa was tested via motility.

**Results:**

There was no effect of OptiPrep™ on motility of fresh and cooled spermatozoa. The supplementation of 1 and 2 % OptiPrep™ increased motility of frozen spermatozoa at 10 min after thawing, while it did not improve motility of spermatozoa at 3 h after thawing in the absence of ATP. During incubation of thawed spermatozoa, the ATP addition protected time-dependent decrease in motility after thawing in OptiPrep™-treated samples. OptiPrep™ had no effect on AMI and MMP in frozen-thawed spermatozoa but combinations of OptiPrep™ and ATP improved MMP in frozen-thawed spermatozoa.

**Conclusions:**

Iodixanol has cryoprotective effects during rat sperm freezing without any toxic effect. Moreover, the combinations of iodixanol and ATP have a beneficial role in maintaining function of frozen-thawed rat spermatozoa for long period of incubation post-thaw.

**Electronic supplementary material:**

The online version of this article (doi:10.1186/s12958-016-0141-5) contains supplementary material, which is available to authorized users.

## Background

The rat has been recognized as one of the most valuable laboratory animals for biomedical and genomic research [1–4]. With the production of a large number of mutant or transgenic rat strains, there is an increasing demand for preservation and distribution of rat lines. Although rats are relatively easy to house and breed, it is expensive to maintain and transport genetically modified strains [5, 6]. For this reason, cryopreservation of gametes and embryos has been developed to maintain genetically valuable materials for an extended period and facilitate transportation of genetic materials at a low cost [7, 8]. In particular, cryopreservation of spermatozoa has been well known as the most efficient way to preserve animal germplasm [9, 10], because of simplicity of epididymal sperm collection, convenience of cryopreservation method and economics for use of a few animals [11, 12]. However, intrauterine insemination (IUI) or in vitro fertilization (IVF) using cryopreserved rat spermatozoa are very limited by the low success of embryo developmental competence and pregnancy rate in most rat strains [10, 12]. Therefore, development of optimal protocols for rat sperm cryopreservation are currently ongoing [12–16].

Iodixanol was originally developed as an x-ray contrast agent [17]. Today, it is also widely applied as a medium for density-gradient centrifugation and cushioned centrifugation technique due to its inert, nontoxic, nonionic characteristics [18–22]. Interestingly, addition of iodixanol to the freezing extender helped maintain membrane integrity and functionality during the cryopreservation process of bovine spermatozoa [23]. It was demonstrated that addition of minor amounts of iodixanol affected the glass transition temperature of the solution and altered the structure of the growing ice crystals. Currently, the use of iodixanol for rat sperm cryopreservation has not been reported. Here, we hypothesized that low level of iodixanol might have cryoprotective properties without exerting toxic effects during rat sperm cryopreservation.

Frozen-thawed viable spermatozoa often cannot maintain viability as long as fresh viable spermatozoa. Thus, the energy production and supplementation are important to support motility of frozen-thawed spermatozoa for successful cryopreservation [14, 24]. Spermatozoa may attain access to eggs by mobilizing metabolic energy production in the form of adenosine triphosphate (ATP) to drive motility [25]. The freezing extender containing exogenous ATP improved the cryosurival of rat epididymal spermatozoa [14]. As modification of the above reports, we thought that ATP can extend lifespan of frozen spermatozoa post-thaw by supplying energy to thawed spermatozoa when frozen spermatozoa are incubated with thawing media containing ATP. Therefore, the aim of this study was to evaluate protective effect of iodixanol on rat spermatozoa during cryopreservation and to determine maintainable effect of ATP on function of frozen rat spermatozoa during incubation after thawing. The goal of this study was to develop an optimized cryopreservation condition for rat spermatozoa using iodixanol and ATP by offering proper condition for enhanced viability of rat spermatozoa after thawing as well as freezing.

## Methods

### Animals

Six sexually mature (12–15 weeks old) male rats (outbred Sprague-Dawley [SD] strain) were used as sperm donors. Rats were housed in conventional rat cages at 20–25 °C in a controlled light environment (10 h dark/14 h light) and provided free access to water and standard rodent chow. The rats were housed in accordance with the policies of the University of Missouri Animal Care and Use Committee and the Guide for the Care and Use of Laboratory Animals.

### Experimental design

The effect of iodixanol (OptiPrep™; 60 % w/v iodixanol in water) was evaluated during cryopreservation of rat spermatozoa. The OptiPrep™ concentrations for this study were 0 % [control], 1, 2, 3 and 4 %. Epididymal spermatozoa was divided into three as following: 1) fresh spermatozoa were incubated with different concentrations of OptiPrep™ for 1 h to test potential toxic effect of iodixanol, 2) spermatozoa were cooled in freezing media containing different concentrations of OptiPrep™ for 1 h and 3 h to determine iodixanol effect on cooling time, 3) spermatozoa were cooled and frozen in freezing media containing different concentrations of OptiPrep™ to evaluate iodixanol effect for cryopreservation.

To check the effect of ATP after thawing, frozen spermatozoa was incubated in media containing 0 mM or 2 mM ATP after thawing. Incubation time was 10 min and 3 h to evaluate the maintaining effect of ATP on sperm function for long incubation. Spermatozoa frozen with different concentrations of OptiPrep™ were thawed and incubated with ATP to evaluate synergistic effect of OptiPrep™ and ATP.

### Sperm collection, freezing and thawing

All chemicals were purchased from Sigma Chemical (St. Louis, MO, USA) unless otherwise stated. Male rats were euthanized by CO_2_ inhalation. The cauda epididymides were excised and placed in 35-mm petri dishes containing HEPES-buffered Tyrode lactate (TL-HEPES) media [26] supplemented with 3 mg/mL bovine serum albumin (BSA) and 0.11 mg/mL pyruvic acid. The cauda epididymides were dissected with a fine scissors to allow spermatozoa to swim out for 10 min at 37 °C. The sperm suspension (fresh spermatozoa) was gently drawn into a plastic Samco transfer pipette (San Fernando, CA, USA) with 2-mm inner diameter and placed in 1.5-mL Eppendorf tube. The initial sperm concentrations were approximately 50 × 10^6^ spermatozoa/mL and only semen with ≥70 % motility was used for this study. The collected spermatozoa were processed individually.

The freezing extender used in this study was the TEST (TES-Tris buffer)-sucrose-egg yolk freezing extender comprised of 25.14 g/L N-tris (hydroxymethyl) methyl-2-aminoethanesulfonicd acid (TES), 13.14 g/L Tris base, 27.34 g/L sucrose, 20 % (vol/vol) egg yolk and 0.75 % (vol/vol) Equex-Paste (Minitüb, Tiefenbach, Germany). The spermatozoa were frozen as previously described [27]. Briefly, one part of fresh spermatozoa was mixed with four parts of freezing extender. The diluted spermatozoa were cooled at 4 °C for 60 min and were loaded into 0.25-mL straws. The straws were placed in liquid nitrogen (LN_2_) vapor for 10 min, plunged into LN_2_ and stored at LN_2_ for at least 7 days. The straws were thawed by holding them in water (37 °C) for 10 s. The 200 μL of thawed spermatozoa were then diluted with 800 μL of HEPES–buffered fertilization media (modified Kreb’s Ringer bicarbonate [mKRB]) at the ratio of 1:4 and incubated at 37 °C for evaluation.

### Iodixanol treatment

Fresh spermatozoa were evaluated for motility after 1 h incubation at 37 °C in TL-HEPES media containing different OptiPrep™ concentrations at the ratio of 1:4 or were kept at 4 °C after addition of freezing extender containing different OptiPrep™ concentration at the ratio of 1:4. The cooled samples were diluted with TL-HEPES (1:4 ratio) 1 and 3 h after cooling initiation, incubated at 37 °C for 10 min, and evaluated for motility. Meanwhile, spermatozoa cooled for 1 h were frozen, diluted with fertilization media (1:4 ratio) after thawing, and incubated for 10 min and 3 h at 37 °C for evaluation of motility. Thawed spermatozoa were also evaluated for acrosomal membrane integrity (AMI) and mitochondrial membrane potential (MMP) at 10 min after thawing.

### ATP treatment

To determine if ATP can play a role as an energy source after thawing, frozen-thawed rat spermatozoa were diluted with fertilization media (1:4 ratio) containing ATP (0 or 2 mM). The samples were processed as described in above section (iodixanol treatment).

### Evaluation of sperm function

#### Sperm motility

Twenty μL of sperm suspension were loaded into 80-μm deep dual-sided chamber (2× CELL; Hamilton Thorne Biosciences) at 37 °C and covered with cover glass. Computer-assisted sperm analysis (CASA, Hamilton Thorne Biosciences Inc, Beverly, MA, USA) system was then used to analyze the motility of rat spermatozoa placed onto the chamber [28]. Total and progressive motilities and average path velocity (VAP) were evaluated in six fields using CASA. The setting parameters and the definition of measured sperm motion parameters for the CASA were: frames per second, 20; duration of tracking time, 0.7 s; medium VAP cutoff, 25.0 μm/s; low VAP cutoff, 5.0 μm/s; count slow as motile, yes.

#### Sperm acrosomal membrane integrity

A PNA-Alexa Fluor 488/propidium iodide (PI) stain (L21409 and L7011, Molecular Probes Inc., Eugene, OR, USA, respectively) was used to determine AMI of rat spermatozoa. Aliquots of 200 μL of diluted frozen spermatozoa (2 ~ 4 × 10^6^/mL spermatozoa) were mixed with 1 μL of 0.2 mg/mL PNA-Alexa Fluor 488 (final concentration, 1 μg/mL) and 1 μL of 200 μM PI (final concentration, 1 μM). The mixture was incubated at 37 °C for 15 min in 5 % CO_2_ and then was analyzed by flow cytometry. Spermatozoa with PNA−/PI−, PNA+/PI− and PNA+ combined PNA+/PI− and PNA+/PI+ were considered to have viable spermatozoa with intact acrosomal membrane, viable spermatozoa with reacted acrosomal membrane and total spermatozoa with reacted/or damaged acrosomal membrane, respectively.

#### Sperm mitochondrial membrane potential (MMP)

The sperm MMP was evaluated using the JC-1 fluorescent dye (Molecular Probes Inc.) as previously described [27]. Aliquots of 200 μL of diluted frozen spermatozoa (2 ~ 4 × 10^6^ spermatozoa/mL) were mixed with 1 μL of 100 μM JC-1 (final concentration, 0.5 μM) and 1 μL of 200 μM PI (final concentration, 2 μM) to exclude dead spermatozoa. The mixture was incubated at 37 °C for 30 min in 5 % CO_2_ and then was analyzed by flow cytometry. The percentage of spermatozoa with viable and high MMP (J_agg_+/PI−) and the Mean J_agg_ fluorescence intensity (J_agg_ MFI) per viable sperm were evaluated. To investigate the population of spermatozoa with high MMP of viable spermatozoa, the ratio of J_agg_ + to viable spermatozoa (%) was calculated as follows: (J_agg_+/PI− [%] ÷ PI− [%]) × 100.

### Flow cytometric analysis

All flow cytometry analyses were performed using a FACSCalibur flow cytometer (Becton Dickinson, San José, CA, USA) equipped with a 15 mW air-cooled 488 nm argon-ion laser and Cell Quest Pro software (Becton Dickinson). A total of 10,000 individual sperm-sized events were selected based on forward and side scatter and were collected at a flow rate of < 400 events/s. FL1 (PNA-Alexa Fluor 488), FL2 (J_agg_) and FL3 (PI) signals were detected through 530 nm, 585 nm and > 670 nm band pass filter, respectively.

### Statistical analysis

Statistical analysis was performed using SPSS software (version 17.0 for Windows; SPSS Inc., Chicago, IL). The Shapiro-Wilk test was utilized for normality analysis of the parameters. To compare the effect following OptiPrep™ concentrations, one-way repeated-measures (RM) analysis of variance (ANOVA) was used in data with a normal distribution and Bonferroni adjustment for multiple comparisons was used for *post hoc* analysis. Otherwise, the non-parametric Friedman test and *post-hoc* analysis with Wilcoxon signed ranks test was conducted with Bonferroni correction for data that was not normally distributed. For comparisons following incubation time and ATP supplementation, paired *t*-test or Wilcoxon signed ranks test was performed according to normality analysis. Statistical significance was set at *P* < 0.05 and all tests were two-tailed. Values were presented as the mean ± standard error of the mean (SEM).

## Results

### The effect of iodixanol on fresh and cooled spermatozoa

**Fig. 1:**
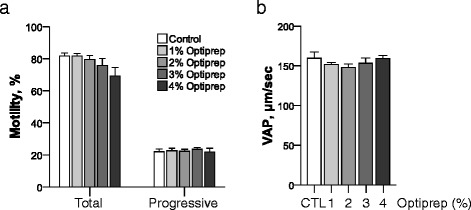
The effect of OptiPrep™ on fresh rat sperm motility. The effect of OptiPrep™ on fresh rat sperm motility was evaluated using total and progressive motility (**a**) and VAP (**b**) after 1 h incubation with different concentrations of OptiPrep™ of fresh spermatozoa at 37 °C. *n* = 6

**Fig. 2:**
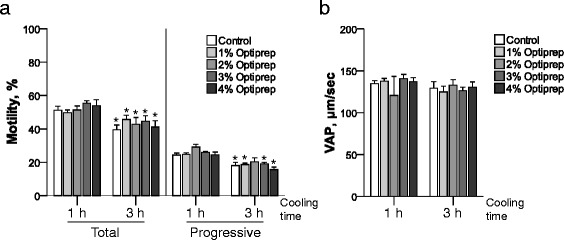
The effect of OptiPrep™ on cooled rat sperm motility. The effect of OptiPrep™ on cooled rat sperm motility was evaluated using total and progressive motility (**a**) and VAP (**b**) 1 and 3 h after initiation of cooling to 4 °C with different concentrations of OptiPrep™ of rat spermatozoa. *P < 0.05 v.s. 1 h after cooling within same iodixanol treatment groups. *n* = 6

When fresh spermatozoa were exposed to TL-HEPES with different concentrations of OptiPrep™, total motility, progressive motility and VAP were not different among samples (*P* > 0.05; Fig. 1). The motility of cooled spermatozoa was also evaluated after 1 and 3 h cooling in different OptiPrep™ concentration (Fig. 2). There were no significant differences in motility of cooled spermatozoa among the different OptiPrep™ concentrations (*P* > 0.05). OptiPrep™ supplementation did not prevent total and progressive motility from decreasing according to cooling time (Fig. 2a). Total motility decreased at 3 h cooling as compared to 1 h cooling and progressive motility also decreased at 3 h cooling relative to 1 h cooling (*P* < 0.05) except for 2 % OptiPrep™ concentration. Duration of cooling had no significant effect on VAP (Fig. 2b). In conclusion, supplementation of OptiPrep™ did not protect decrease in sperm motility caused by longer periods of cooling except for progressive motility shown in 2 % OptiPrep™ concentration. These data suggest that iodixanol had no protective effect for cooling process and no adverse effects on motility of fresh and cooled rat spermatozoa as well.

### The effect of iodixanol and ATP on motility of frozen-thawed spermatozoa

**Fig. 3:**
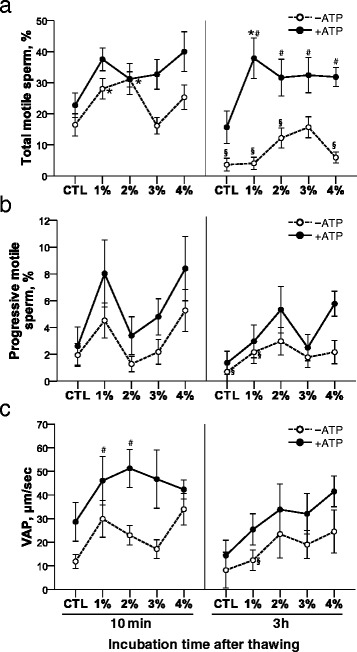
The effect of OptiPrep™ and ATP treatment on post-thaw motility of rat spermatozoa. **a** Total motile sperm, **b** Progressive motile sperm, **c** Average path velocity (VAP), *P < 0.05 v.s. control (CTL) within same incubation time and ATP treatment groups (comparison among OptiPrep™ concentrations), ^#^ *P* < 0.05 v.s. ATP-free within same incubation time and OptiPrep™ groups (comparison between ATP-free and ATP-supplemented groups), and ^§^ *P* < 0.05 v.s. 10 min within same OptiPrep™ and ATP treatment groups (comparison between 10 min and 3 h incubation time). *n* = 6

Samples frozen with different OptiPrep™ concentrations were evaluated for motility after thawing and incubating in the presence or absence of ATP (Fig. 3). When the samples were incubated for 10 min in the absence of ATP post-thaw, the 1 and 2 % OptiPrep™ concentrations were superior to the other groups in total sperm motility (*P* < 0.05; Fig. 3a). However, there was no difference in total motility among OptiPrep™ concentrations at 3 h incubation under ATP-free media. Total sperm motility of 0, 1, 2 and 4 % OptiPrep™ showed remarkable reduction for 3 h incubation as compared to 10 min incubation, while only the 3 % OptiPrep™ appeared to prevent reduction in total motility for long incubation time post-thaw in the absence of ATP (Fig. 3a).

Interestingly, total motility was maintained for 3 h incubation in presence of ATP, without any differences between 10 min and 3 h incubation in all OptiPrep™ concentrations (Fig. 3a). In particular, total motility of OptiPrep™-treated groups increased in ATP-supplemented media as compared to ATP-free media at 3 h incubation (Fig. 3a). The incubation with ATP-supplemented media after thawing prevented decrease in rat sperm motility caused by longer incubation after freezing-thawing. The 1 % OptiPrep™ concentration showed a high percentage of total motility among different OptiPrep™ concentrations for 3 h incubation with ATP supplementation, indicating that combinations of iodixanol and ATP can be beneficial system to increase and maintain the number of motile rat spermatozoa even in longer incubation post-thaw.

There were no differences in progressive motility and VAP among OptiPrep™ concentrations at 10 min and 3 h incubation post-thaw (Fig. 3b and c). ATP improved VAP of 1 and 2 % OptiPrep™ concentrations at 10 min incubation post-thaw (Fig. 3c), supporting synergistic effect of iodixanol and ATP. Three h incubation decreased progressive sperm motility in control and 1 % OptiPrep™ and VAP in 1 % OptiPrep™ as compared to 10 min incubation post-thaw in absence of ATP, while it did not show a decrease in progressive sperm motility and VAP in all groups in the presence of ATP. In conclusion, low concentrations (1 and 2 %) of OptiPrep™ enhanced sperm motility with ATP-supplementation.

### The effect of iodixanol and ATP on AMI of frozen-thawed spermatozoa

**Fig. 4:**
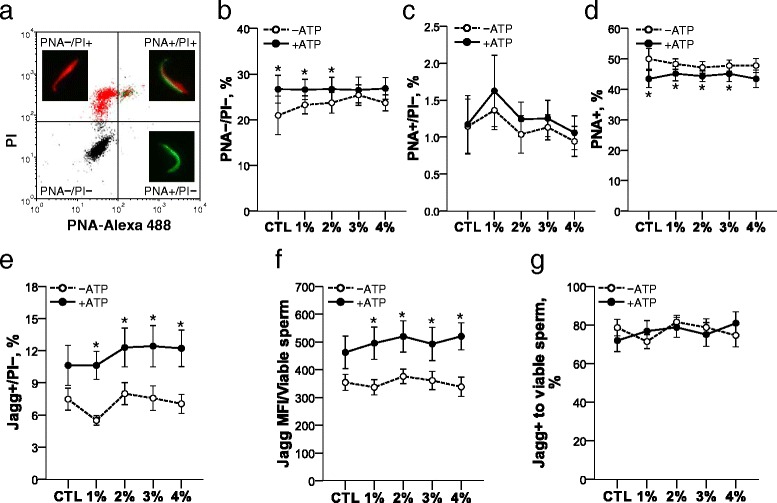
The effect of OptiPrep™ and ATP treatment on post-thaw AMI and MMP of rat spermatozoa. Frozen-thawed spermatozoa were stained with PNA-Alexa 488/PI (**a**) and the percentages of PNA−/PI− (**b**), PNA+/PI− (**c**) and PNA+/PI+ (**d**) were analyzed with flow cytometry. Using JC-1/PI staining, viable spermatozoa with high MMP (**e**), Mean J_agg_ fluorescence intensity (J_agg_ MFI) per viable sperm (**f**), ratio of spermatozoa with high MMP of viable spermatozoa (**g**) were evaluated for MMP. PNA−/PI−: viable and acrosome-intact, PNA+/PI−: viable and acrosome-reacted, PNA+/PI+: dead and acrosome-reacted/or damaged, PNA−/PI+: dead, PNA+: total acrosome-reacted/or damaged. *P < 0.05 v.s. ATP-free within OptiPrep™ groups (comparison between ATP-free and ATP-supplemented groups). *n* = 6

Sperm acrosomal status after freezing-thawing was divided into 4 categories depending on PNA/PI staining patterns using flow cytometry and epifluorescence microscope (Fig. 4a). There was no significant difference in the percentages of PNA−/PI− (viable and intact acrosome), PNA+/PI− (viable and reacted acrosome) and PNA+ (total reacted/or damaged acrosome) spermatozoa among samples frozen under different OptiPrep™ concentrations regardless of ATP presence (Fig. 4b–d). However, when the frozen-thawed samples were diluted with ATP-supplemented media, the percentage of PNA−/PI− spermatozoa increased in control, 1 and 2 % OptiPrep™ as compared to dilution with ATP-free media (Fig. 4b). Meanwhile, the percentage of PNA+/PI− spermatozoa did not show differences in all groups by ATP treatment (Fig. 4c) and the percentage of PNA+ spermatozoa decreased in control, 1, 2 and 3 % OptiPrep™ by dilution with ATP-supplemented media as compared to dilution with ATP-free media (Fig. 4d). The OptiPrep™ had no effect on acrosome membrane while ATP can maintain stability of acrosome membrane in frozen-thawed rat spermatozoa.

### The effect of iodixanol and ATP on MMP of frozen-thawed spermatozoa

Similar to effect on acrosome membrane of OptiPrep™ and ATP, there was no significant difference in MMP among samples frozen with different OptiPrep™ concentrations regardless of ATP (Fig. 4). However, ATP supplementation after thawing increased the percentage of Jagg+/PI− (viable and high MMP) spermatozoa and improved MMP of viable spermatozoa in groups treated with OptiPrep™ (Fig. 4e and f). Mean 75 % of viable frozen spermatozoa were Jagg + spermatozoa without significant differences following OptiPrep™ and ATP treatment (Fig. 4g). The combination of OptiPrep™ and ATP remarkably improved MMP function of frozen-thawed rat spermatozoa.

## Discussion

Recently, it has been demonstrated that iodixanol is beneficial when added to a freezing extender for maintaining membrane integrity and functionality during the cryopreservation process of bovine and ram semen [23, 29]. Additionally, iodixanol contributed to preserving sperm motility after the freeze-thaw cycle. This study aimed to determine if iodixanol exhibited similar protective effects on rat spermatozoa during cryopreservation.

Supplementation of 5 % OptiPrep™ protected post-thaw progressive motility, membrane integrity, acrosomal integrity and morphological damages in ram semen [29] and 2.5 % OptiPrep™ was superior for post-thaw motility in bovine spermatozoa [23]. In our study, low concentrations (1 or 2 %) of OptiPrep™ were superior to the higher concentration groups 10 min after thawing under ATP-free condition for total motility. The possible mechanisms that iodixanol protects spermatozoa during cryopreservation are attributed to an elevation of glass transition temperature and alteration of ice crystal formation in a non-colligative fashion, resulting in optimal environment for freezing [23]. Iodixanol alters ice crystal formation into intricate nets of dendrites in a concentrations-dependent manner during freezing [23]. Loose net of dendrites formed by lower concentrations of OptiPrep™ helped increase the cryoprotective effect on rat spermatozoa as compared to more compact net of dendrites formed by higher concentrations used in bovine and ram spermatozoa cryopreservation. Spermatozoa vary in size and shape according to species, which may be critical factors susceptible to structural changes of freezing condition by iodixanol. Falciform-shaped head and long tail of rat spermatozoa may serve as one of factors responsible for a better protective effect in lower concentrations of OptiPrep™ as compared to another species.

OptiPrep™ had a cryoprotective effect on freezing, not cooling, as shown in improved motility of 1 and 2 % OptiPrep™ at 10 min after thawing in our study. OptiPrep™ did not help preventing the decrease in total motility for longer storage of post-thaw as seen in 3 h incubation after thawing. Inevitable decrease in motility following time still occurred in cryopreserved rat spermatozoa in spite of OptiPrep™ treatment, with highly susceptible characteristics of rat spermatozoa to various external environments. In fact, when incubating for 3 h after thawing, sharp declines of total motility were observed even in 1 and 2 % OptiPrep™ groups, which showed an increase in total sperm motility at the early time point (10 min) after thawing in absence of ATP. Based on these results, long manipulation times consumed for AMI and MMP measurement may make difficult to determine the effect of OptiPrep™ on cryopreserved rat spermatozoa.

To overcome decreases in rat sperm motility caused by elapsed time after thawing, our second goal was to sustain motility of frozen-thawed rat spermatozoa by continuous supply of ATP. Both intracellular and extracellular ATP molecules play key roles in sperm function [30]. Intracellular ATP is the main energy source driving sperm motility [30]. Extracellular ATP produces several downstream effects that improve sperm motility by increasing calcium levels or by activating cAMP signal transduction pathways [14]. However, there were no reports describing the effect of ATP on spermatozoa in thawing media as far as we know. This is first study to determine if supplement of ATP into thawing media can maintain the function of rat spermatozoa survived from cryodamage.

In this study, ATP supplementation to thawing medium prevented the decrease in total motility observed in groups incubated with ATP-free thawing medium for 3 h (Fig. 3a). ATP supplemented-Optiprep™ groups showed improvement in total motility compared to ATP-free-Optiprep™ groups at 3 h incubation of thawing (Fig. 3a). In particular, total motility of the 1 % Optiprep™-treated group was superior to that of all other groups at 3 h incubation after thawing in ATP-containing media. This finding indicates that exogenous ATP can maintain sperm motility for longer periods of storage after thawing and combination of ATP and iodixanol can have a synergistic effect on enhancement of sperm motility. Therefore, ATP treatment with iodixanol may be beneficial during assisted fertilization by maintaining and extending activity of functional spermatozoa after freezing-thawing. In fact, extracellular ATP treatment of fresh mouse spermatozoa enhanced IVF success throughout alteration of motility by increased intracellular calcium level [30].

ATP supplementation also led to improvement in MMP in all groups treated with Optiprep™ as compared to ATP-free condition. Moreover, the combination of ATP and Optiprep™ had a strong synergistically effect in enhancing MMP function of frozen rat spermatozoa after thawing. However, considering that ATP increased AMI of frozen rat spermatozoa even in Optiprep™-free group as well as 1 and 2 % Optiprep™ groups, ATP seems to improve AMI of frozen rat spermatozoa alone regardless of Optiprep™ treatment.

The effect of extracellular ATP treatment on sperm acrosome is controversial. Extracellular ATP was reported to increase acrosomal exocytosis (AE) in human and bovine fresh spermatozoa [31, 32] and did not affect AE in mouse fresh spermatozoa [30], while it increased the proportion of intact acrosomes during freezing as well as rat fresh sperm [14]. In the current study, ATP supplementation inhibited AE and increased acrosomal membrane integrity of cryopreserved rat spermatozoa in some Optiprep™ groups as well as control group without Optiprep™ treatment, indicating that ATP may affect capacity of rat spermatozoa to fertilize oocytes. The mechanisms that ATP influences on AMI will be studied in rat spermatozoa in the future.

Surgical intra uterine insemination (IUI) using rat sperm frozen in 2 % OptiPrep™ resulted in pregnancies and live fetuses (Additional file 1: Table S1). While 9 out of 10 (90 %) rats become pregnant using fresh spermatozoa, 4 out of 11 (36 %) rats became pregnant using frozen-thaw sperm treated with 2 % OptiPrep™ (Additional file 1: Table S1). Although we did not compare the pregnancy and delivery rate between the absence and presence of OptiPrep™, this study led to a possibility for fertilization and pregnancy using frozen-thawed rat spermatozoa. Further testing on OptiPrep™ will be needed to overcome low fertilization and pregnancy rate of frozen rat spermatozoa [12, 33].

## Conclusion

Iodixanol supplementation with low concentrations to freezing extender increased post-thaw motility. ATP protected spermatozoa from the decrease in motility by post-thaw time and improved AMI & MMP when combined with iodixanol. This study suggests that combinations of iodixanol and ATP may lead successful freezing and fertilization of rat spermatozoa by cryoprotective effect of iodixanol during freezing and maintenance ability of ATP after thawing.

## Additional file

Additional file 1:
**The additional file included method and data for “Developmental competence of frozen-thaw rat spermatozoa in the presence of 2 % Optiprep™ after surgical intra uterine insemination.”** For more detail, see Additional file 1. (PDF 78 kb)

## Abbreviations

AE: acrosomal exocytosis
AMI: acrosomal membrane integrity
ATP: adenosine 5′-triphosphate
BSA: bovine serum albumin
CASA: Computer-assisted sperm analysis
FL1: channel detecting green fluorescence
FL2: channel detecting orange fluorescence
FL3: channel detecting red fluorescence
IUI: intrauterine insemination
IVF: in vitro fertilization
Jagg: JC-1 aggregated form
Jagg+: highly MMP potential
JC-1: 5, 5′, 6, 6′- tetrachloro-1, 1′, 3, 3′-tetraethyl-benzimidazolylcarbocyanine iodide
MFI: mean fluorescence intensity
MMP: mitochondrial membrane potential
PI: propidium iodide
TES: N-tris (hydroxymethyl) methyl-2-aminoethanesulfonicd acid
