# Supplementary material for: Post-thaw ATP supplementation enhances cryoprotective effect of iodixanol in rat spermatozoa
Source: Reprod Biol Endocrinol. 2016 Jan 29;14:5. doi: 10.1186/s12958-016-0141-5 (PMC4731941; doi:10.1186/s12958-016-0141-5)
Supplement: Additional file 1: — The additional file included method and data for “Developmental competence of frozen-thaw rat spermatozoa in the presence of 2 % Optiprep™ after surgical intra uterine insemination.” For more detail, see Additional file 1. (PDF 78 kb) [file 12958_2016_141_MOESM1_ESM.pdf]

Additional file 1

Submitted to ***Reproductive Biology and Endocrinology***

“Research Articles” category

Full title: Post-thaw ATP supplementation enhances cryoprotective effect of iodixanol in rat spermatozoa

Suhee Kim[1], Sarah Hooper[1], Cansu Agca[1] and Yuksel Agca[1,*]

[1]Department of Veterinary Pathobiology, College of Veterinary Medicine, University of Missouri-Columbia, Columbia, Missouri 65211, USA

*Corresponding author.

Yuksel Agca, DVM, MS, PhD

University of Missouri

College of Veterinary Medicine

1600 East Rollins Street, Room W191

Columbia, MO 65211

Phone: (573) 884-0311

E-mail: agcay@missouri.edu

Keywords: ATP, Cryopreservation, rat spermatozoa, Iodixanol

Additional Materials and Methods

Animals

Twenty one sexually mature female rats (8-10 weeks old) were used for artificial insemination (AI).

Surgical Artificial Insemination

Intra uterine insemination (IUI) was performed with sperm that was frozen using extender contained 2% OptiPrep™, which improved sperm evaluation parameters. In order to prepare pseudo pregnant females for artificial insemination (AI), 8-10 week-old SD rats were synchronized by using intraperitoneal injections of 40 µg Des-Gly^{10}, D-Ala^{6} LHRH ethylamide acetate salt hydrate and subsequently mated with vasectomized males 4 day after LHRH injection. For AI, the pseudo pregnant females were anesthetized by injection of Ketamine (4.17 mg/100 g body weight) and Xylazine (0.83 mg/ 100g body weight) mixture. The fat pad surrounding the ovary were gently grasped with a tissue forceps and retracted/extracted until exposing the utero-tubal junction using standard surgical procedure. Both frozen–thawed and fresh sperm samples were diluted in Hepes-buffered mKRB containing 0.4% (w/v) BSA until insemination. About 50 ul sperm suspension (containing 3-4×10^{6} spermatozoa) were loaded into a ~200 µm inner diameter glass transfer pipette, and the pipette was inserted into the uterine lumen and gently deposited. On the morning of day 21-22, the inseminated females were euthanized and were dissected to confirm pregnancy and counting numbers of live fetuses.

Table S1 Developmental competence of frozen-thaw rat spermatozoa in the presence of 2% Optiprep™ after surgical intra uterine insemination

Treatment	No. Recipients	No. Pregnant (%)	No. total live fetus	No. live fetus per recipient
Fresh	10	9 (90)^a	83	9.2^a
Frozen-thawed	11	4 (36)^b	15	3.75^b

Values within a column with different superscripts differ significantly ($P < 0.05$).
